# Prognostic significance of lncRNA AP004608.1 in prostate cancer

**DOI:** 10.3389/fonc.2022.1017635

**Published:** 2022-09-29

**Authors:** Wei Li, Runze Zhou, Bo Sun, Xin Jin, Yuan Chen, Xuefen Xu

**Affiliations:** ^1^ Jiangsu Key Laboratory for Pharmacology and Safety Evaluation of Chinese Materia Medica, School of Pharmacy, Nanjing University of Chinese Medicine, Nanjing, China; ^2^ Institute of Traditional Chinese medicine (TCM)-Related Comorbid Depression, School of Chinese Medicine & School of Integrated Chinese and Western Medicine, Nanjing University of Chinese Medicine, Nanjing, China; ^3^ Department of Pharmacy, Suzhou Municipal Hospital, The Affiliated Suzhou Hospital of Nanjing Medical University, Suzhou, China; ^4^ Department of Pharmacology, School of Medicine and Holistic Integrative Medicine, Nanjing University of Chinese Medicine, Nanjing, China

**Keywords:** prostate cancer, AP004608.1, survival, meta-analysis, prognostic

## Abstract

This study aimed to screen and determine the value of AP004608.1 expression as a biomarker for Prostate cancer (PCa) survival. We investigated the expression and prognosis of AP004608.1 through bioinformatics analysis. Low AP004608.1 expression predicted favorable Overall survival (OS) and Progression-free survival (PFS) in PCa patients, according to the Cancer Genome Atlas (TCGA) database. Cox regression demonstrated that low AP004608.1 expression were in-dependent biomarkers for OS. Moreover, Gene Expression Omnibus (GEO) database was utilized to verify the prognostic role of AP004608.1 in PCa, and the similar results were reached. A meta-analysis revealed that low AP004608.1 expression was closely relevant to better OS. AP004608.1 could constitute a promising prognostic biomarker, and probably plays an important role in PCa.

## Introduction

PCa is the second most common solid tumor in men and the fifth cause of cancer mortality (1, 2). The incidence and mortality rates of shows an extreme geographical variation, and increases progressively with the age of the worldwide population (2–6). In addition to the above two unmodifiable factors, the dysregulation of hormonal pathways due to several modifiable environmental factors, leads to an increased high-grade risk (7–10). Although the death rate from has decreased as diagnostic and therapeutic procedures have improved, the early diagnosis and prognosis of individual patients varied substantially due to the tumor’s heterogeneity (11–14). Hence, finding a novel biomarker with high accuracy is critical for achieving individualized PCa diagnosis and prognosis assessment.

The current clinical approaches in diagnosis include digital rectal examination (DRE), prostate-specific antigen (PSA) measurement and prostate biopsies (15). However, the effectiveness of DRE can only reach 5% -30%, and is contingent on the experience and skill of the examiner (16). PSA is an organ but not a cancer-specific marker, its sensitivity ranges between 67.5% and 80%, therefore, about 20–30% of could not be diagnosed (3). Liquid biopsy based on circulating tumor cells (CTCs), extracellular vesicles (EVs), circulating tumor DNA (ctDNA) and RNA (ctRNA) has emerged as an attractive and promising strategy complementary to invasive tissue biopsy to guide diagnosis and treatment (3, 17–19). What’s more, liquid biopsy showed a significant potential to modify PCa management since ability to represent comprehensive information and follow-up the progression of PCa.

Long non-coding RNA (lncRNA), a class of biologically functional non-coding RNAs longer than 200 bases, has become a hotspot in the molecular biology in PCa. The abnormal expression of lncRNA is closely related to the progression, metastasis and prognosis of PCa (20–23). AP004608.1 is a newly discovered lncRNA, and is abnormally expressed in PCa, pancreatic and lung adenocarcinoma (24–27). However, the potentially target mRNAs, the clinical and prognostic significance of AP004608.1 in PCa is still unknown, and its functional role in PCa has never been documented.

In this study, we first screened and determined AP004608.1 expression as a biomarker for PCa survival. We used bioinformatics to analyze RNA sequencing (RNA-Seq) data from tissue gene expression profiles in the TCGA database, mined new genes closely related to prognosis, analyzed the relation of low AP004608.1 expression and OS and PFS in PCa patients, and assessed the prognostic significance of AP004608.1 expression as an in-dependent biomarker for OS (28–31). Then, we validated the prognostic role of AP004608.1 using GEO database. In addition, we performed a meta-analysis and revealed that low AP004608.1 expression was closely relevant to better OS.

## Materials and methods

### Differentially expressed genes mining from public databases

489 PCa tissue samples and 51 normal tissue samples mRNA data were downloaded from the TCGA database (https://portal.gdc.cancer.gov/) and PCa from the GEO database (https://www.ncbi.nlm.nih.gov/geo/) Expression profile microarray data (GSE6956) was downloaded for a total of 89 cases. The Wilcox Test method was used to screen for differential genes in tumor and normal tissues, with the screening condition: |log FC>2| (log FC=log2 ^mean tumor sample expression^ - log2 ^mean normal sample^, log FC>0 indicates up-regulated genes, log FC<0 indicates down-regulated genes), significance of corrected gene expression differences P<0.05. Using R scripts, the common differentially expressed genes were screened.

### Independent prognostic genes screening

Differential genes were filtered using the KM method (discrete algorithm) and the cox (continuous algorithms) in turn. The survival genes were the differential genes that had both derived P < 0.05 and a standard deviation < 0.1. Then, P values were determined by comparing survival genes to other clinical indicators (age, T-stage, M-stage, and N-stage) and survival genes were considered independent prognostic genes if P<0.05. Moreover, Roc Filter was used to assess the accuracy of genes as prognostic genes. 0.7≤AUC<1 was used as the standard in this study.

### The expression analysis and clinical relevance of AP004608.1

TCGA data, including gene expression data (HTSeq-FPKMA) for 551 cases, methylation data (IIIumina Human Methylation 450) for 553 cases, survival data, clinical indicators (age, T-stage, M staging, N staging), and tumor progression-free survival (PFS) data, were downloaded using the UCSC-XENA tool (https://xena.ucsc.edu/). The P of single gene difference between normal and tumor samples was calculated (P<2.22×10^-16^) and plotted in box plots; OS plots of overall survival curves for AP004608.1 high and low expression groups were plotted; subject work characteristic (ROC) curves were plotted to interpret AP004608.1 prognostic factors accuracy of patient survival over large spans (3, 5, 10 years).

### Meta-analysis

On the relationship between the AP004608.1 and PCa, data from the GEO database and the TCGA database were used. The overall prognostic significance of the AP004608.1 in PCa was assessed using meta-analysis. To investigate the link between the expression of the AP004608.1 and the prognosis of PCa patients, combined hazard ratio (HR) and 95% confidence intervals (CIs) were determined using R-language scripts. The Q (I2) test was used to analyze heterogeneity between the two datasets, and in this study, I2 = 0%, P>0.05 heterogeneity was low, so a fixed-effect model was chosen for combination and forest map.

### Statistical analysis

R was used to conduct all of the analyses (v.4.1.0). The charts were analyzed using “GraphPad” program. For the study of statistical paired data, the Wilcoxon signed-rank test and the t-test were utilized. A statistically significant difference was defined as P<0.05.

## Results

### PCa patients’ prognosis-related differential gene screening

The RNA-Seq database of 489 columns of PCa tissue samples and 51 columns of normal prostate tissue samples was downloaded from the TCGA database and screened for differential genes using R, yielding 3664 genes that were significantly differentially expressed between PCa tissue and normal prostate tissue, including 2220 genes with upregulated expression and 1444 genes with downregulated expression. Kaplan-Meier and Cox survival analysis revealed that 41 genes were significantly differentially expressed between PCa tissue and normal prostate tissue (P<0.05), as shown in **Table 1**. Five independent prognostic genes, IGHV7-81, AP004608.1, AP000844.2, SNORD6 and LRRC31, were further screened by R script based on the survival gene screening. Meanwhile, the AUC in ROC curves were used to verify the accuracy of their prognostic genes. The AUC for AP004608.1, which reached 0.8525, was the highest of these five prognostic genes, indicating that this gene was also more accurate as a prognostic gene (**Table 2**). Finally, we found that the difference between AP004508.1 gene and two variables of T-stage and N-stage were more significant, LRRC31 only differed significantly from T-stage, and the remaining three genes were not significantly different from the clinical correlation variables, so we chose AP004608.1 as the -related differential gene for further study.

**Table 1:** 41 genes that were significantly differentially expressed between PRAD tissue and normal prostate tissue.

gene	KM	HR	HR.95L	HR.95H	coxPvalue
SNORD46	0.044792	1.394128	1.006728	1.930604	0.045465
AC138956.1	0.031999	3.165423	1.386651	7.225974	0.006215
AL645608.3	0.026605	41.2258	4.810913	353.2733	0.000691
AURKB	0.04318	1.263061	1.045166	1.526381	0.01564
PKMYT1	0.027562	1.838296	1.371072	2.464737	4.72E-05
GPC2	0.049016	2.898814	1.513391	5.552513	0.00133
ASF1B	0.035175	1.203636	1.074301	1.348541	0.001395
SGO1	0.037142	3.568178	1.361585	9.35079	0.009657
AC010624.4	0.01758	1.340393	1.115476	1.610661	0.001772
C3orf35	0.018038	5.528871	1.588852	19.23931	0.007194
IGHV7-81	0.008431	2.899995	1.712845	4.909944	7.40E-05
MAPK8IP2	0.014488	1.133093	1.012141	1.268499	0.030045
BAIAP2L2	0.032791	1.065545	1.018032	1.115275	0.006375
AL645608.6	0.035309	1.262609	1.06743	1.493477	0.006497
CDK1	0.01826	1.243942	1.103242	1.402586	0.000365
IMPDH1P8	0.035092	10.18362	1.963488	52.81725	0.005721
SNHG12	0.044664	1.338685	1.039511	1.723962	0.023807
AP004608.1	0.006289	1.152994	1.044242	1.273072	0.004856
MSH5	0.037415	4.738355	1.215802	18.46684	0.024993
PAQR6	0.017479	1.052628	1.011039	1.095929	0.012641
PRR22	0.00465	1.381309	1.004302	1.899843	0.046993
AC124944.3	0.015796	3.706836	1.556956	8.825319	0.003073
CDCA5	0.043104	1.387009	1.162191	1.655317	0.000288
LIME1	0.044941	2.145148	1.441313	3.192688	0.000169
AC007387.1	0.003325	2.146145	1.070192	4.303845	0.031473
AC078883.3	0.016575	1.57E-05	3.34E-10	0.742876	0.043999
PIMREG	0.033851	1.738084	1.255111	2.406906	0.000875
AP000844.2	0.045875	1.075191	1.047675	1.10343	4.23E-08
HOXB-AS2	0.014017	7.624699	1.086907	53.48761	0.040972
CPT1B	0.041149	3.58249	1.809376	7.093185	0.000251
AGAP13P	0.034999	3.647851	1.572694	8.461164	0.002572
SNORD6	0.014834	1.380355	1.117654	1.704804	0.002766
AL513329.1	0.021964	41.35817	2.514181	680.34	0.009181
RRM2	0.011236	1.261978	1.055328	1.509092	0.010767
KIFC1	0.024302	1.242046	1.073857	1.436576	0.003502
EZH2	0.035529	1.703925	1.302593	2.228908	0.000101
CTAGE7P	0.048204	19.96943	1.454885	274.096	0.025057
PRDX3P2	0.029859	2.559621	1.130084	5.797498	0.024251
SKA1	0.033251	2.471289	1.59848	3.820673	4.70E-05
LRRC31	0.024816	1.096245	1.012889	1.18646	0.022765
NCAPH	0.046546	1.383665	1.034959	1.849858	0.028387

**Table 2:** 5 independent prognostic genes.

gene	AUC	HR	HR.95L	HR.95H	pvalue
AP004608.1	0.852469	1.15262	1.020065	1.302399	0.022686
IGHV7-81	0.85006	5.231614	1.538964	17.78455	0.008037
SNORD6	0.842508	1.399056	1.106651	1.768723	0.004999
AP000844.2	0.817361	1.070025	1.037912	1.103132	1.34E-05
LRRC31	0.758501	1.09895	1.011208	1.194305	0.026249

### The clinical and prognostic value of AP004608.1 expression according to TCGA database

**Figure 1:**
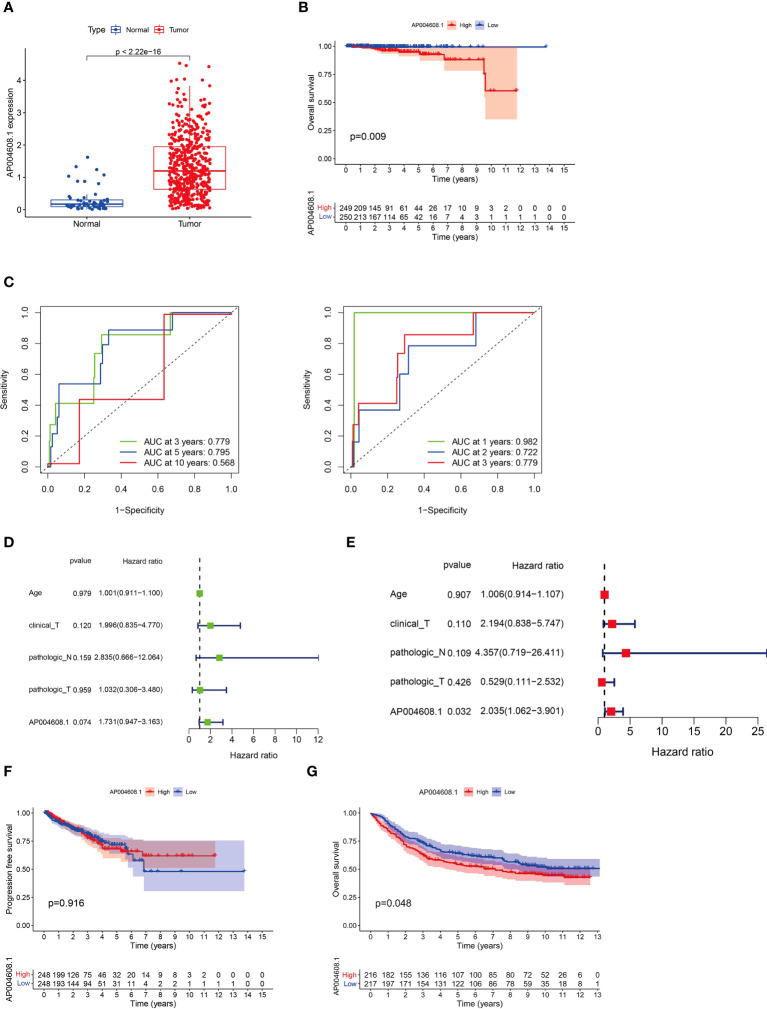
The clinical and prognostic value of AP004608.1 expression according to TCGA database. **(A)** AP004608.1 mRNA is highly expressed in PCa tissues in TCGA dataset. **(B)** The OS curves for the high and low expression groups of the AP004608.1 based on TCGA database. **(C)** The ROC curves that using the AP004608.1 showed the accuracy of predicting patient survival for large spans (3, 5 and 10 years) and small spans (1, 2 and 3 years) respectively. **(D)** Univariate screening of relevant prognostic genes for age, T-stage, M-stage, N-stage and AP004608.1expression. **(E)** Multifactorial screening of relevant prognostic genes for age, T-stage, M-stage, N-stage and AP004608.1 expression. **(F)** The PFS curves for the high and low expression groups of the AP004608.1. **(G)** The OS curves for the high and low expression groups of the AP004608.1 based on GEO database.

The RNA-Seq of the AP004608.1 from the TCGA database (51 cases in the normal prostate group and 489 cases in PCa tissues) revealed that the mRNA expression of the AP004608.1 was low in normal prostate tissues and highly expressed in PCa tissues, with a statistically significant difference (P<2.22×10^-16^) as shown in **Figure 1A**. **Figure 1B** showed the OS curves for the high and low expression groups of the AP004608.1, with statistically significant variations in OS between the two groups (P<0.05). The ROC curves showed that using the AP004608.1, the accuracy of predicting patient survival for large spans (3, 5 and 10 years) was 0.779, 0.795, and 0.568, respectively (**Figure 1C**), while the accuracy of predicting patient survival for small spans (1, 2 and 3 years) was 0.982, 0.722, and 0.779, respectively (**Figure 1C**). Univariate screening of relevant prognostic genes based on P<0.05 and HR >1 was performed for age, T-stage, M-stage, N-stage and AP004608.1expression, and then multifactorial analysis was performed for age, T staging, N staging, and AP004608.1 expression, and it was discovered that P<0.05 for AP004608.1, indicating that AP004608.1 could be used as a key prognostic gene in PCa independently of other clinical features (**Figures 1D, E**). As illustrated in **Figure 1F**, PFS curves for the high and low expression groups of the AP004608.1 were plotted, and there was no significant difference in PFS between the two groups (P=0.916).

The GEO database’s AP004608.1 RNA-Seq (PCa expression profile microarray data of 89 cases) revealed that AP004608.1 mRNA was lowly expressed in normal prostate tissues and highly expressed in PCa tissues, with a statistically significant difference (P<0.05) between the two groups, as shown in **Figure 1G**. The AP004608.1 was also found to be a critical prognostic gene for prostate cancer treatment.

### The correlation analysis of AP004608.1 expression and clinical indicators

**Figure 2:**
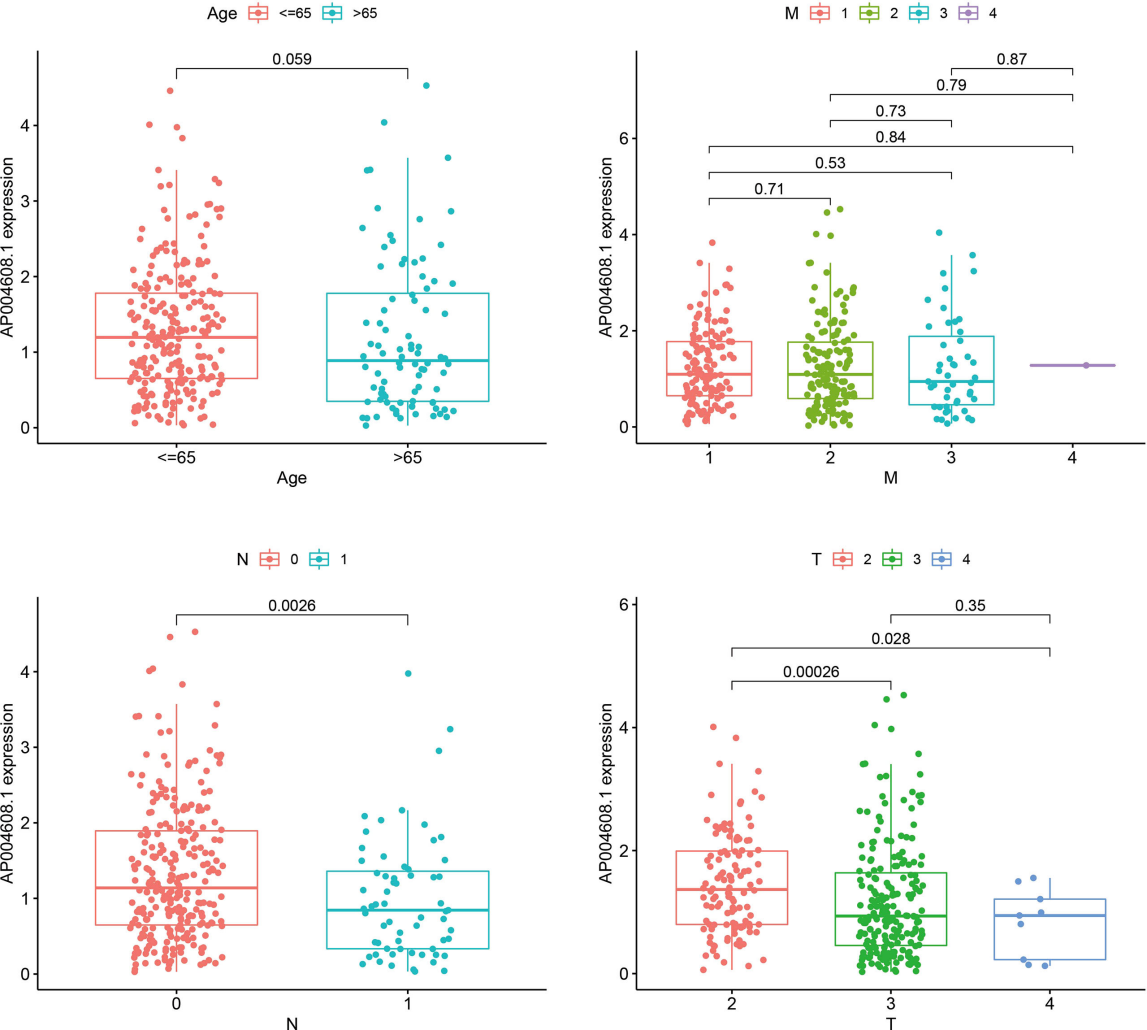
The correlation analysis of AP004608.1 expression and clinical indicators. The correlation analysis of AP004608.1 expression and Age, M stage, T stage, and N stage.

Age, M stage, T stage, and N stage were used to group four factors, and the association between each component and AP004608.1 expression was calculated. If P<0.05, the factor was connected with AP004608.1 expression, and if P>0.05, it was not correlated with AP004608.1 expression. Age, M-stage, and T-stage III/IV were not connected with AP004608.1 expression (P>0.05), as shown in **Table 3** and **Figure 2**. T-stage II/III, II/IV, and N-stage were correlated with AP004608.1 expression (P<0.05).

**Table 3:** AP004608.1 gene expression and clinical indicators correlation analysis.

Covariates	Type	Total	High	Low	Pvalue
Age	<=65	240 (73.17%)	130 (79.27%)	110 (67.07%)	0.0179
Age	>65	88 (26.83%)	34 (20.73%)	54 (32.93%)	
M	1	130 (39.63%)	66 (40.24%)	64 (39.02%)	0.6618
M	2	150 (45.73%)	76 (46.34%)	74 (45.12%)	
M	3	47 (14.33%)	21 (12.8%)	26 (15.85%)	
M	4	1 (0.3%)	1 (0.61%)	0 (0%)	
N	0	265 (80.79%)	138 (84.15%)	127 (77.44%)	0.161
N	1	63 (19.21%)	26 (15.85%)	37 (22.56%)	
T	2	116 (35.37%)	72 (43.9%)	44 (26.83%)	0.0044
T	3	203 (61.89%)	89 (54.27%)	114 (69.51%)	
T	4	9 (2.74%)	3 (1.83%)	6 (3.66%)	
expression	High	164 (50%)	164 (100%)	0 (0%)	0.001
expression	Low	164 (50%)	0 (0%)	164 (100%)	

### Meta-analysis and predictive performance of AP004608.1 expression

**Figure 3:**
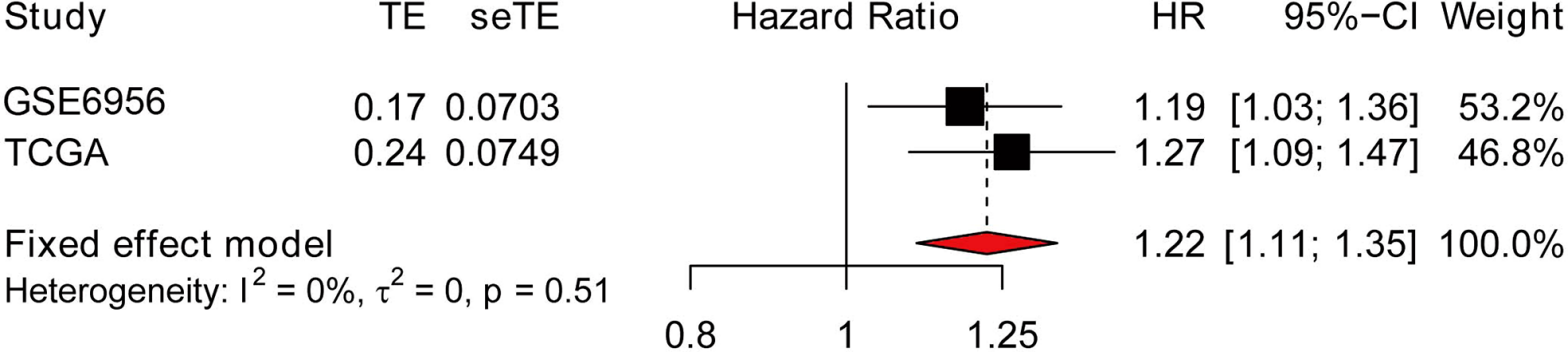
Forest plot of AP004608.1 expression with overall prognosis in PCa patients from TCGA and GEO datasets.

Meta-analysis was used to analyze the importance of the AP004608.1 in the overall prognosis of PCa patients using data from the TCGA and GEO datasets. To analyze the connection between AP004608.1 expression and the prognosis of PCa patients, the combined HR and 95% C were determined. This gene has a combined HR of 1.22 (>1), indicating that it is a high-risk gene for PCa development and has a good link with prostate cancer prognosis (**Figure 3**).

## Discussion

In our study, we analyzed the clinical and prognostic role of AP004608.1 expression in PCa according to TCGA database. For the first time, we discovered the strongly negative association between AP004608.1 expression and OS of PCa. We found that AP004608.1 expression was closely associated with a series of significant features, including histological type and molecular type (24, 27, 32). Cox regression models established the critical role of low AP004608.1 expression in the favorable prognosis of patients with PCa. In addition, this study also verified the AP004608.1 expression in the GEO database and confirmed the important role of AP004608.1 expression in the prognosis of PCa patients. Meta-analysis of 551 prostate cancer patients from the TCGA database and 89 prostate cancer patients from the GEO database revealed that AP004608.1 expression is an independent predictive factor in PCa patients’ OS. Our analyses confirmed the close correlation AP004608.1 expression and clinical indicators (Age, T-stage, M-stage, N-stage) of PCa, which is to say, low AP004608.1 expression was closely relevant to better OS. Collectively, our analyses emphasized that AP004608.1 is a promising biomarker for predicting prognosis of patients with PCa.

The identification of several novel biomarkers in tumor tissues, serum, and even urine was facilitated by the advances in chip technology and next-generation high-throughput sequencing (NGS). However, only a few biomarkers have been approved for use by the US Food and Drug Administration (FDA) (PSA in 1994, PHI in 2012, and PCA3 in 2012). due to its limited tumor specificity, more effective diagnostic/prognostic indicators for PCa are still lacking in clinical practice (22, 23). Thus, for the clinical diagnosis and treatment of prostate cancer, it is critical to discover the markers that are associated with PCa diagnosis and prognosis (24). Among these novel biomarkers, lncRNA has received more and more attention and has become the hotspot of PCa research. For example, the abnormal expression of lncRNA T1 and OIP5-AS1 is related to PCa progression through AKT/NF-κB signaling and ferroptosis resistance respectively (33, 34). lncRNA NEAT1 and T6 were reported to promotes bone metastasis in PCa (35, 36). However, there is currently insufficient evidence that which lncRNA can function as a biomarker of PCa diagnosis and prognosis. AP004608.1 is a newly discovered lncRNA, and is abnormally expressed in PCa, pancreatic and lung adenocarcinoma (13–16). We based the AP004608.1 expression in PCa tissues and normal prostate tissues on the clinical diagnosis and prognosis of PCa on the basis of RNA-Seq differential gene screening. According to a series of bioinformatics investigations, Cox regression modeling and Meta-analysis of 640 PCa patients from the TCGA and GEO databases, the results herald that AP004608.1 is expected to be a new target and prognostic factor for PCa treatment.

It is worth noting that this study also has certain limitations, such as: Only the TCGA database contains PFS information, and the relationship between AP004608.1 gene expression and PFS, thus it could not be verified in the GEO database. In addition, in this study, through GO and KEGG enrichment analysis, the role of AP004608.1 gene expression in PCa was preliminarily discussed. However, the AP004608.1 gene modification (such as methylation) and the potential mechanism linking AP004608.1 gene expression and modification with PCa still needs further biomedical experiments to verify.

## Conclusion

We examined the sequencing data of a large sample of PCa using bioinformatics tools, and exposed the potential therapeutic and prognostic relevance of the AP004608.1 in PCa. Low AP004608.1 expression predicts favorable prognosis in PCa patients. Hence, AP004608.1 could act as a promising biomarker in PCa patients. Our study provided hints and a foundation for further research into the gene’s biological function and mechanism of action.

## Data availability statement

The datasets presented in this study can be found in online repositories. The names of the repository/repositories and accession number(s) can be found in the article/supplementary material.

## Author contributions

Author contribution: XXand WL designed the study and interpreted data; WL, RZ and BS analyzed the data and wrote the manuscript; RZ, BS, XJ and YC collected the data; WL, RZ, BS, XJ and YC analyzed the data. XX and WL revised the manuscript. All authors contributed to the article and approved the submitted version.

## Funding

This study was supported by the National Natural Science Foundation of China (Nos. 81703542) and the Natural Science Foundation of Nanjing University of Chinese Medicine(NZY81703542).

## Acknowledgments

We gratefully acknowledge the assistance of Prof. Min Hong and Jie Zheng (Nanjing University of Chinese Medicine) for helpful discussions on topics related to this work.

## Conflict of interest

The authors declare that the research was conducted in the absence of any commercial or financial relationships that could be construed as a potential conflict of interest.

## Publisher’s note

All claims expressed in this article are solely those of the authors and do not necessarily represent those of their affiliated organizations, or those of the publisher, the editors and the reviewers. Any product that may be evaluated in this article, or claim that may be made by its manufacturer, is not guaranteed or endorsed by the publisher.

## Abbreviations

PCa, prostate cancer; OS, overall survival; PFS, progression-free survival; TCGA, the cancer genome atlas; GEO, gene expression omnibus; PSA, prostate specific antigen; RNAseq, RNA sequencing; ROC, receiver operating characteristic; HR, hazard ratio.

## References

[1] Sung H Ferlay J Siegel RL Laversanne M Soerjomataram I Jemal A . Global cancer statistics 2020: GLOBOCAN estimates of incidence and mortality worldwide for 36 cancers in 185 countries. Ca-Cancer J Clin (2021) 71(3):209–49. doi: 10.3322/caac.21660 33538338

[2] Gandaglia G Leni R Bray F Fleshner N Freedland SJ Kibel A . Epidemiology and prevention of prostate cancer. Eur Urol Oncol (2021) 4(6):877–92. doi: 10.1016/j.euo.2021.09.006 34716119

[3] Crocetto F Russo G Di Zazzo E Pisapia P Mirto BF Palmieri A . Liquid biopsy in prostate cancer management-current challenges and future perspectives. Cancers (2022) 14(13):3272–89. doi: 10.3390/cancers14133272 PMC9265840 35805043

[4] Pernar CH Ebot EM Wilson KM Mucci LA . The epidemiology of prostate cancer. Csh Perspect Med (2018) 8(12):a030361–79. doi: 10.1101/cshperspect.a030361 PMC6280714 29311132

[5] Mazzone E Preisser F Nazzani S Tian Z Bandini M Gandaglia G . The effect of lymph node dissection in metastatic prostate cancer patients treated with radical prostatectomy: A contemporary analysis of survival and early postoperative outcomes. Eur Urol Oncol (2019) 2(5):541–8. doi: 10.1016/j.euo.2018.10.010 31411992

[6] Mustafa M Abu Rass H Yahya M Hamdan K Eiss Y . Primary metastatic prostate cancer between prognosis or adequate/proper medical therapy. World J Surg Oncol (2021) 19(1):5–10. doi: 10.1186/s12957-020-02111-3 PMC7783967 33397422

[7] Gacci M Russo GI De Nunzio C Sebastianelli A Salvi M Vignozzi L . Meta-analysis of metabolic syndrome and prostate cancer. Prostate Cancer P D (2017) 20(2):146–55. doi: 10.1038/n.2017.1 28220805

[8] Vidal AC Oyekunle T Howard LE De Hoedt AM Kane CJ Terris MK . Obesity, race, and long-term prostate cancer outcomes. Cancer-Am Cancer Soc (2020) 126(16):3733–41. doi: 10.1002/cncr.32906 32497282

[9] Olivas A Price RS . Obesity, inflammation, and advanced prostate cancer. Nutr Cancer (2021) 73(11-12):2232–48. doi: 10.1080/01635581.2020.1856889 33287566

[10] Crocetto F Pandolfo SD Aveta A Martino R Trama F Caputo VF . A comparative study of the Triglycerides/HDL ratio and pseudocholinesterase levels in patients with bladder cancer. Diagnostics (2022) 12(2):431–44. doi: 10.3390/diagnostics12020431 PMC8871224 35204522

[11] Kohaar I Petrovics G Srivastava S . A rich array of prostate cancer molecular biomarkers: Opportunities and challenges. Int J Mol Sci (2019) 20(8):1813–31. doi: 10.3390/ijms20081813 PMC6515282 31013716

[12] Khoo A Liu LY Nyalwidhe JO Semmes OJ Vesprini D Downes MR . Proteomic discovery of non-invasive biomarkers of localized prostate cancer using mass spectrometry. Nat Rev Urol (2021) 18(12):707–24. doi: 10.1038/s41585-021-00500-1 PMC8639658 34453155

[13] Rahmani E Zaitlen N Baran Y Eng C Hu DL Galanter J . Sparse corrects for cell type heterogeneity in epigenome-wide association studies. Nat Methods (2016) 13(5):443. doi: 10.1038/Nmeth.3809 27018579 PMC5548182

[14] Ciccarese C Massari F Iacovelli R Fiorentino M Montironi R Di Nunno V . Prostate cancer heterogeneity: Discovering novel molecular targets for therapy. Cancer Treat Rev (2017) 54:68–73. doi: 10.1016/j.ctrv.2017.02.001 28231559

[15] Nguyen-Nielsen M Borre M . Diagnostic and therapeutic strategies for prostate cancer. Semin Nucl Med (2016) 46(6):484–90. doi: 10.1053/j.semnuclmed.2016.07.002 27825428

[16] Ragsdale JW Halstater B Martinez-Bianchi V . Prostate cancer screening. Primary Care (2014) 41(2):355. doi: 10.1016/j.pop.2014.02.009 24830612

[17] Dathathri E Isebia KT Abali F Lolkema MP Martens JWM Terstappen LWMM . Liquid biopsy based circulating biomarkers in metastatic prostate cancer. Front Oncol (2022) 12:863472. doi: 10.3389/fonc.2022.863472 35669415 PMC9165750

[18] Tulpule V Morrison GJ Falcone M Quinn DI Goldkorn A . Integration of liquid biopsies in clinical management of metastatic prostate cancer. Curr Oncol Rep (2022) 24(10):1287–98. doi: 10.1007/s11912-022-01278-0 PMC9474724 35575959

[19] Bin Riaz I Wang L Kohli M . Liquid biopsy approach in the management of prostate cancer. Transl Res (2018) 201:60–70. doi: 10.1016/j.trsl.2018.05.004 29936077 PMC6631037

[20] Mitobe Y Takayama K Horie-Inoue K Inoue S . Prostate cancer-associated lncRNAs. Cancer Lett (2018) 418:159–66. doi: 10.1016/j.canlet.2018.01.012 29330107

[21] Zhang Y Huang YX Wang DL Yang B Yan HY Lin LH . LncRNA DSCAM-AS1 interacts with YBX1 to promote cancer progression by forming a positive feedback loop that activates FOXA1 transcription network. Theranostics (2020) 10(23):10823–37. doi: 10.7150/thno.47830 PMC7482804 32929382

[22] Wu GL Hao C Qi XL Nie JQ Zhou WM Huang J . LncRNA SNHG17 aggravated prostate cancer progression through regulating its homolog SNORA71B via a positive feedback loop. Cell Death Dis (2020) 11(5):393–406. doi: 10.1038/s41419-020-2569-y PMC7245601 32447342

[23] Hua JT Ahmed M Guo HY Zhang YZ Chen SJ Soares F . Risk SNP-mediated promoter-enhancer switching drives prostate cancer through IncRNA T19. Cell (2018) 174(3):564. doi: 10.1016/j.cell.2018.06.014 30033362

[24] Chen G Qin XP Wang Y Gao BY Ling MA Yin WJ . Expression status and prognostic value of autophagy-related lncRNAs in prostate cancer. Cell Cycle (2022) 21(16):1684–96. doi: 10.1080/15384101.2022.2065149 PMC9302510 35414328

[25] Zhu WJ Gao WZ Deng YY Yu X Zhu HW . Identification and development of long non-coding RNA associated regulatory network in pancreatic adenocarcinoma. Oncotargets Ther (2020) 13:12083–96. doi: 10.2147/Ott.S265036 PMC7699307 33262608

[26] Wang JX Yin XJ Zhang YQ Ji XM . Identification and validation of a novel immune-related four-lncRNA signature for lung adenocarcinoma. Front Genet (2021) 12:639254. doi: 10.3389/fgene.2021.639254 33708243 PMC7940686

[27] Zhao F Wang M Zhu J . Hypoxia-related lncRNAs to build prognostic classifier and reveal the immune characteristics of EGFR wild type and low expression of PD-L1 squamous and adenocarcinoma NSCLC. Cancer Med-Us (2021) 10(17):6099–113. doi: 10.1002/cam4.4126 PMC8419766 34250747

[28] El-Zeiny AM Khdery G Gad AA . Hyperspectral based approach to investigate topsoil characteristics of different taxonomic units of El-fayoum depression. Egypt J Remote Sens (2022) 25(2):405–15. doi: 10.1016/j.ejrs.2022.02.007

[29] Holmgren HG Stockdale L Gale M Coyne SM . Parent and child problematic media use: The role of maternal postpartum depression and dysfunctional parent-child interactions in young children. Comput Hum Behav (2022) 133:107293–9. doi: 10.1016/j.chb.2022.107293

[30] He X Wang ND Li Z Zhang S Yao Z Xie XX . Network pharmacology and GEO database-based analysis of sini powder in the prevention of depression among shift workers. All Life (2022) 15(1):74–87. doi: 10.1080/26895293.2021.2019130

[31] Le JNF Jawad K Feygin Y Lohr WD Creel L Jones VF . Examination of US national rates of emergency department visits and hospitalizations for depression and suicidal behaviors after the release of the 13 reasons why Netflix series by demographic characteristics. J Affect Disord (2022) 311:508–14. doi: 10.1016/j.jad.2022.05.116 35636515

[32] Hua S Xie ZW Wang WH Wan Z Chen M Zhao S . Identification and validation of a novel immune-related lncRNA signature for bladder cancer. Front Oncol (2021) 11:704946. doi: 10.3389/fonc.2021.704946 34322391 PMC8311739

[33] Shang ZQ Yu JP Sun LB Tian J Zhu SM Zhang BY . LncRNA T1 activates AKT and NF-kappa b signaling in castration-resistant prostate cancer by regulating the PHLPP/FKBP51/IKK alpha complex. Nucleic Acids Res (2019) 47(8):4211–25. doi: 10.1093/nar/gkz108 PMC6486551 30773595

[34] Zhang YY Guo SQ Wang S Li XJ Hou DK Li HZ . LncRNA OIP5-AS1 inhibits ferroptosis in prostate cancer with long-term cadmium exposure through miR-128-3p/SLC7A11 signaling. Ecotox Environ Safe (2021) 220:220–28. doi: 10.1016/j.ecoenv.2021.112376 34051661

[35] Wen SM Wei YL Zen C Xiong W Niu YJ Zhao Y . Long non-coding RNA NEAT1 promotes bone metastasis of prostate cancer through N6-methyladenosine. Mol Cancer (2020) 19(1):171–88. doi: 10.1186/s12943-020-01293-4 PMC7733260 33308223

[36] Lang CAD Yin C Lin KY Li Y Yang Q Wu ZQ . m(6)A modification of lncRNA T6 promotes bone metastasis in prostate cancer through IGF2BP2-mediated IGF1R mRNA stabilization. Clin Transl Med (2021) 11(6):e426–48. doi: 10.1002/ctm2.426 34185427 PMC8181202

